# Incidence of active mycobacterial infections in Brazilian patients with chronic inflammatory arthritis and negative evaluation for latent tuberculosis infection at baseline - A longitudinal analysis after using TNFα blockers

**DOI:** 10.1590/0074-02760150235

**Published:** 2015-11

**Authors:** Carina Mori Frade Gomes, Maria Teresa Terreri, Maria Isabel de Moraes-Pinto, Cássia Barbosa, Natália Pereira Machado, Maria Roberta Melo, Marcelo Medeiros Pinheiro

**Affiliations:** 1 Universidade Federal de São Paulo, Escola Paulista de Medicina, Disciplina de Reumatologia, São Paulo, SP, Brasil; 2 Universidade Federal de São Paulo, Escola Paulista de Medicina, Departamento de Pediatria, Setor de Reumatologia Pediátrica, São Paulo, SP, Brasil; 3 Universidade Federal de São Paulo, Escola Paulista de Medicina, Departamento de Pediatria, Setor de Infectologia Pediátrica, São Paulo, SP, Brasil

**Keywords:** mycobacterial infection, tuberculosis, incidence, latent tuberculosis infection, chronic arthritis, TNFα blockers, tuberculin skin test

## Abstract

Several studies point to the increased risk of reactivation of latent tuberculosis infection (LTBI) in patients with chronic inflammatory arthritis (CIAs) after using tumour necrosis factor (TNF)*α* blockers. To study the incidence of active mycobacterial infections (aMI) in patients starting TNF *α* blockers, 262 patients were included in this study: 109 with rheumatoid arthritis (RA), 93 with ankylosing spondylitis (AS), 44 with juvenile idiopathic arthritis (JIA) and 16 with psoriatic arthritis (PsA). All patients had indication for anti-TNF *α* therapy. Epidemiologic and clinical data were evaluated and a simple X-ray and tuberculin skin test (TST) were performed. The control group included 215 healthy individuals. The follow-up was 48 months to identify cases of aMI. TST positivity was higher in patients with AS (37.6%) than in RA (12.8%), PsA (18.8%) and JIA (6.8%) (p < 0.001). In the control group, TST positivity was 32.7%. Nine (3.43%) patients were diagnosed with aMI. The overall incidence rate of aMI was 86.93/100,000 person-years [95% confidence interval (CI) 23.6-217.9] for patients and 35.79/100,000 person-years (95% CI 12.4-69.6) for control group (p < 0.001). All patients who developed aMI had no evidence of LTBI at the baseline evaluation. Patients with CIA starting TNF *α* blockers and no evidence of LTBI at baseline, particularly with nonreactive TST, may have higher risk of aMI.

The World Health Organization (WHO) estimates that one-third of the global population is infected by *Mycobacterium tuberculosis*. Brazil is one of the major countries responsible for most cases of tuberculosis (TB) in the world. Incident rates range from 30 cases per 100,000 inhabitants in the South and Central-West Regions to 50 cases per 100,000 individuals in the North, Northeast and Southeast Regions (Conde et al. 2009).

It is believed that 3-5% of the Brazilian population has some type of chronic inflammatory arthritis (CIA), including rheumatoid arthritis (RA), ankylosing spondylitis (AS), psoriatic arthritis (PsA) and juvenile idiopathic arthritis (JIA), and 30-50% of them will need to use an immunobiologic agent (Senna et al. 2004, Mota et al. 2012).

However, some studies have shown greater rates of active mycobacterial infections (aMI) after the introduction of these medications (Fonseca et al. 2006, Patkar et al. 2008, Dixon et al. 2010). Most of these cases are associated with the reactivation of latent TB infection (LTBI) and usually occur in the first six months of tumour necrosis factor alpha (TNFα) blocker use, being lower in patients exposed to etanercept (ETN) when compared to users of monoclonal antibodies infliximab (IFX) or adalimumab (ADA) (Patkar et al. 2008). A recent review on the incidence of granulomatous infections after treatment with biological agents reported to the US Food and Drug Administration showed that the incidence of TB varied from 35-144 cases per 100,000 patients treated, depending on the medication used (Wallis et al. 2004). In Brazil, there is no published data on the incidence of infectious granulomatous diseases related to the use of anti-TNFα agents.

Thus, given the increasing use of TNFα blockers for the treatment of various CIAs, as well as registration, notification and increased severity of mycobacterial infections, it is mandatory to screen and identify cases of LTBI before treatment with these agents (Mangini & Melo 2003, Ormerod et al. 2005). After the adoption of these measures, including a 6-9-month regimen with isoniazid (INH), the incidence of active TB infection decreased significantly (Reino et al. 2007, Aggarwal et al. 2009).

The objective of this study was to prospectively evaluate the incidence of aMI in patients with active CIAs after using TNFα inhibitors.

## PATIENTS, MATERIALS AND METHODS

A total of 262 patients were included in this study, of which there were 109 with a diagnosis of RA (Arnett et al. 1988), 93 with AS (Dougados et al. 1991), 44 with JIA (Petty et al. 2004) and 16 with PsA (Taylor et al. 2006). All patients had disease activity classified as moderate to severe according to the specific indexes of each disease and indication for the initiation of anti-TNFα therapy (Mota et al. 2012, Sampaio-Barros et al. 2013). The control group consisted of 215 healthy and asymptomatic individuals, matched for age, sex and social class, from the database of primary care units of metropolitan area from São Paulo, Brazil, and administered by the Federal University of São Paulo (UNIFESP).

Adopted exclusion criteria of the study included the inability to answer questions from the questionnaires, as well as patients with the overlap of other autoimmune rheumatic diseases or neoplasms and those with contraindication to anti-TNFα therapy (Motta et al. 2012, Sampaio-Barros et al. 2013). In addition, all of them were negative for viral infection caused by human immunodeficiency virus, hepatitis B and C.

Anthropometric, socioeconomic, demographic and clinical data were evaluated in all study subjects, as well as details on symptoms related to mycobacterial infection and epidemiologic background for TB, such as personal and family history, prior treatment and professional contact. The specific characteristics of each disease were studied through structured questionnaire, of which included the Disease Activity Score 28 (DAS-28) (Prevoo et al. 1995), Bath Ankylosing Spondylitis Disease Activity Index (BASDAI) (Garret et al. 1994), Psoriasis Area and Severity Index (PASI) (Langley & Ellis 2004) and activity score for patients with JIA (Wallace et al. 2004). Furthermore, the presence of associated diseases and concomitant and current treatment was verified, especially glucocorticoid steroids (GCs) and disease-modifying antirheumatic drugs (DMARDs), which can interfere with the positivity of the tuberculin skin test (TST).

Additionally, a simple chest radiograph was performed on all patients, which was evaluated by two experienced rheumatologists. In inconclusive cases, a pulmonologist and a radiologist were consulted to establish consensus. If the case remained inconclusive, a high resolution computed tomography of the chest was performed. The TST was conducted in patients and controls, complying with existing international recommendations, with the inoculation of 0.1 mL (2 UT) of purified protein derivative (PPD) RT-23, applied intradermally in the middle third of the anterior region of the left forearm, approximately 8 cm below the elbow flexion, with reading after 72 h. TST was considered to be positive if the size of the indurated area measured ≥ 5 mm and these patients received treatment for LTBI with INH 10 mg/kg/day (children) or 300 mg/day (adults) for six months.

During 48 months, the patients were followed up by clinical and laboratory evaluation to identify cases of aMI. Adverse event (AE) is defined as any medical occurrence not initially foreseen in a study participant that emerged after anti-TNFα therapy (ICH 2010).

*Statistical analysis* - The numerical data are presented in mean ± standard deviation and the categorical variables in percentage values. The Student´s*t* test was used for the comparison of means between groups. For the comparison of nonnumeric data, ANOVA and the Tukey range test were chosen. Tests of association between the positivity of the TST and clinical, demographic and epidemiological variables were made through the chi-square test of association and logistic regression models were made with LTBI as a dependent variable to identify associated risk factors. The Statistical Package for Social Science software v.15.0 was used for all statistical analysis. The level of significance was defined as p < 0.05.

The subjects were informed about the study and agreed to participate, signing a written term of free and informed consent. The research protocol was examined and approved by the Ethical and Research Committee of UNIFESP/EPM (1478/09).

## RESULTS

The initial demographic data of 218 adult patients and 251 healthy controls (HCs) are presented in Table I. Both groups were matched for age, sex, skin colour and socioeconomic condition.

**TABLE I:** Initial demographic data of 218 adult patients with chronic inflammatory arthropathies and 251 healthy controls (HCs)

	Patients with CIA^*a*^ ( n = 218)	HCs ( n = 251)	p
Age (years)	51.5 ± 10.4	50.2 ± 9.7	0.12
Sex (n (%)]			
Female	128 (58.7)	151 (60.2)	0.17
Male	90 (41.3)	100 (39.8)	0.21
Skin colour (n (%)]			
White	123 (56.4)	150 (59.8)	0.09
Nonwhite	95 (43.6)	101 (40.2)	0.1
Socioeconomic class (n (%)]			
A/B	40 (18.3)	55 (21.9)	0.2
C/D/E	178 (81.7)	196 (78.1)	0.21

*a*: with the exclusion of children and adolescents; CIA: chronic inflammatory arthritis. Student's *t*test, ANOVA.

Most patients had long disease duration (time since diagnosis), as well as important activity and disease severity (Table II). DMARDs and diseases which may cause some compromise of cellular immunity can also be viewed in that Table. GCs were being used by the majority of patients with RA, but not by those with AS and PsA. The current average dosage of prednisone and methotrexate (MTX) in patients with RA was 8.7 ± 5.3 mg/day and 22.8 ± 3.5 mg/week, respectively. Inadequate response (63.3%) was the main reason for discontinuing the use of MTX, followed by hepatic and/or gastrointestinal toxicity (36.7%) in all patients with active CIA.

**TABLE II:** Initial demographic and clinical characteristics of 262 patients with chronic inflammatory arthropathies according to diagnosis

	Patients with chronic inflammatory arthropathies ( n = 262)			
	RA ( n = 109)	AS ( n = 93)	PA ( n = 16)	JIA ( n = 44)
Age (years)	54.7 ± 12.1	43.6 ± 10.2^*a*^	56.2 ± 9.3	13.2 ± 4.1
Disease duration (years)	13.1 ± 5.2	12.6 ± 7.3	10.7 ± 5.6	6.1 ± 4.3
Female sex (n (%)]	94 (86.2)^*a*^	25 (26.9)	9 (56.2)	20 (45.5)
Disease activity				
DAS-28	5.7 ± 1.2	-	4.9 ± 1.1	-
BASDAI	-	4.8 ± 1.6	-	-
PASI	-	-	10.4 ± 4.7	-
ILAR (n (%)]	-	-	-	44 (100)
Severity of disease (HAQ/CHAQ)	1.5 ± 0.7	1.2 ± 0.5	1.1 ± 0.4	1.9 ± 0.96^*b*^
Current concomitant medication (n (%)]				
NSAIDs	30 (27.5)	58 (62.4)^*a*^	6 (37.5)	35 (79.5)
Glucocorticoids	82 (75.2)^*a*^	12 (12.9)	1 (6.2)	19 (43.2)
Methotrexate	74 (67.9)	39 (41.9)^*a*^	9 (56.2)	36 (81.8)
Leflunomide	58 (53.2)	2 (2.2)^*a*^	10 (62.5)	9 (20.5)
Diabetes mellitus (n (%)]	14 (12.8)	4 (4.3)	3 (18.8)^*a*^	0 (0)

*a:* p < 0.05; *b: *only Children Health Assessment Questionnaire (CHAQ); AS: ankylosing spondylitis; BASDAI: Bath Ankylosing Spondylitis Disease Activity Index; DAS-28: Disease Activity Score 28; HAQ: Health Assessment Questionnaire; ILAR: International League of Associations for Rheumatology; JIA: juvenile idiopathic arthritis; NSAIDs: nonsteroidal antiinflammatory drugs; PA: psoriatic arthritis; PASI: Psoriasis Area Severity Index; RA: rheumatoid arthritis. Student's *t* test, ANOVA, Tukey test.

Epidemiologic, personal, family and professional data related to TB were identified in approximately 10% of adult patients, with no significant difference between the diseases (Table III).

**TABLE III:** Epidemiologic data of 262 patients with chronic inflammatory arthropathies according to diagnosis

	Patients with chronic inflammatory arthropathies ( n = 262) n (%)			
	RA ( n = 109)	AS ( n = 93)	PA ( n = 16)	JIA ( n = 44)
With any epidemiology data	11 (10.1)	12 (12.9)	3 (18.7)	0 (0)
Personal	3 (27.3)	4 (33.3)	1 (33.3)	0 (0)
With previous treatment	2 (66.7)	2 (50)	1 (100)	0 (0)
Without previous treatment	1 (33.3)	2 (50)	0 (0)	0 (0)
Family history	6 (54.5)	6 (50)	2 (66.7)	0 (0)
Past (> 12 months)	5 (83.3)	4 (66.7)	2 (66.7)	0 (0)
Recent (< 12 months)	1 (16.7)	2 (33.3)	0 (0)	0 (0)
Professional	2 (18.2)	2 (16.7)	0 (0)	0 (0)

AS: ankylosing spondylitis; JIA: juvenile idiopathic arthritis; PA: psoriatic arthritis; RA: rheumatoid arthritis. Student's*t* test, ANOVA, Tukey test.

The vast majority of patients had no abnormality on chest radiography at baseline evaluation. Only 16 adults (7.4%) showed any alteration on chest radiography in the opinion of the two independent evaluators, of which the majority had RA. The principal radiographic findings are summarised in Table IV, in which all were inconclusive and did not suggest a prior granulomatous infection, including apical calcifications. Of these, it was necessary to perform a thorax tomography [computerised tomography (CT) scan] in only three (18.8%) patients in order to clarify the first radiographic findings. However, the CT scan findings were inconclusive and there was no evidence of active TB infection in none of them.

**TABLE IV:** Chest radiographic findings of 262 patients with chronic inflammatory arthropathies according to diagnosis

	Patients with chronic inflammatory arthropathies ( n = 262) n (%)			
	RA ( n = 109)	AS ( n = 93)	PA ( n = 16)	JIA ( n = 44)
Without alterations	99 (90.8)	88 (94.6)	15 (93.8)	44 (100)
With alterations	10 (9.2)	5 (5.4)	1 (6.2)	0 (0)
Pleural thickening	2 (20)	2 (40)	0 (0)	0 (0)
Calcified nodule	3 (30)	3 (60)	1 (100)	0 (0)
Reticulointerstitial infiltrate	4 (40)	0 (0)	0 (0)	0 (0)
Fibrosis	4 (40)	0 (0)	0 (0)	0 (0)

AS: ankylosing spondylitis; JIA: juvenile idiopathic arthritis; PA: psoriatic arthritis; RA: rheumatoid arthritis. Student's*t* test, ANOVA, Tukey test.

Before beginning the TNFα blockers, the positivity of the TST, as a screening method for LTBI, was higher in patients with AS than in other populations with CIA (p < 0.001). In the control group, TST positivity was 32.7% (Table V). Considering only patients with positive TST, it was observed that the most of them had an indurated area above 10 mm, regardless of diagnosis and no significant association with current dosage of GCs or DMARDs. It is noteworthy emphasising that all patients with a TST ≥ 5 mm received LTBI treatment with INH for six months, according to Brazilian's recommendations. No serious AE related to INH was reported in 56 (25.7%) adult patients that used it. Eight (14.3%) patients, especially with RA (62.5%) (p = 0.04), presented mild-intensity paraesthesia in the lower limbs. Peripheral neuropathy improved with vitamin B-complex supplementation. However, in two patients, leflunomide had also to be discontinued. Mild to moderate transitory transaminase elevation was observed in eight (3.7%) patients and improved after the termination of LTBI treatment. None of the patients had to suspend the medication (INH) due to AEs.

**TABLE V:** Positivity of tuberculin skin test (TST) in the initial evaluation of latent tuberculosis infection in 262 patients with chronic inflammatory arthropathies and 251 healthy controls (HCs)

	Patients with chronic inflammatory arthropathies ( n = 262) n (%)							
TST	RA ( n = 109)	AS ( n = 93)	PA ( n = 16)	JIA ( n = 44)	HCs ( n = 251)	p
Positivity	14 (12.8)^*a*^	35 (37.6)	3 (18.8)^*a*^	2 (4.5)^*a*^	82 (32.7)	< 0.001
5.0-9.9 mm	4 (28.6)	9 (25.7)	0 (0)	1 (50)	29 (35.4)	0.09
> 10 mm	10 (71.4)	26 (74.3)	3 (18.8)	1 (50)	53 (64.6)	0.08

*a*: significant lower proportion of positivity; AS: ankylosing spondylitis; JIA: juvenile idiopathic arthritis; PA: psoriatic arthritis; RA: rheumatoid arthritis. ANOVA, Tukey test.

Analysing individually each CIA, there was no significant association of TST positivity with sex, age, skin colour, socioeconomic condition, body mass index, time of diagnosis, activity of the disease (DAS-28, BASDAI, PASI, International League of Associations for Rheumatology, Health Assessment Questionnaire), concomitant disease, use of nonsteroidal antiinflammatory drugs, DMARDs or GCs, radiographic alterations or any other epidemiological aspect for TB. However, after analysing the differences between the groups of CIA, it was found that patients with RA had significantly lower response to PPD than those with AS and PsA. In this scenario, the main clinical variables associated were more advanced age, female sex and the use of GCs, MTX and leflunomide (p = 0.03). It is important to underscore that values above 10 mm were more likely to be found in all CIAs and HCs than values between 5-9.9 mm at a ratio of 2-3:1 (p = 0.08).

After an average follow-up period of 31 months, nine new cases of aMI (3.4%) were observed, of which two (22.2%) were in patients with RA. The remaining six adult patients (66.7%) were those with AS. In all those cases, active infection by*M. tuberculosis* (3 patients with pulmonary disease, 2 with miliary involvement and 1 lymphatic TB) occurred less than six months after initiation of anti-TNFα therapy. In children with JIA, one child was treated due to probable pulmonary disease with compatible respiratory symptoms, nonreactive TST, but with altered chest tomography and positive Elispot assay after 24 months of ADA use.

The definitive diagnosis of infection was possible in all adult patients. The positivity of acid-fast bacilli was established through microbiological laboratory exams (Ziehl-Nielsen stain, identification, culture and drug susceptibility testing of sputum, bronchoalveolar lavage, pleural fluid or lymph node aspirate) and, in some cases, histopathological exams. Table VIillustrates the incidence of aMI according to CIA in adults and in children and adolescents, considering those exposed and not exposed. In control group, the aMI's incidence rate was 35.79/100,000 person-years (95% confidence interval 12.4-69.6) (p < 0.001).

**TABLE VI:** Incidence of active mycobacterial infections according to chronic inflammatory arthropathies and age group

	Adults				Children and adolescents
	Total ( n = 262)	RA ( n = 109)	AS ( n = 93)	PA ( n = 16)	JIA ( n = 44)
Incidence of active mycobacterial infections (100,000 person-years; 95% confidence interval)	86.93; 23.6-217.9	37.04; 25.5-51.7	130.32; 97.8-213.2	0	22.53; 13.9-61.7

AS: ankylosing spondylitis; JIA: juvenile idiopathic arthritis; PA: psoriatic arthritis; RA: rheumatoid arthritis.

IFX was being used in all adult patients, with the exception of the case of Hansen's disease, which had used IFX for 24 months and was using ADA for 15 days. Although it is known that Hansen's disease is of long duration, there was a temporal relationship between the exchange agent and the appearance of skin lesions. These aspects did not allowed to clarify the exact mechanism by which this latent granulomatous infection was disorganised by ADA or IFX (Freitas et al. 2010). Most had good evolution after treatment; however, four (44.4%) patients had therapeutic difficulties, necessitating change of the treatment regimen due to resistance or relapse of the disease. No death was observed. It is worth to point out that in the first medical evaluation, there was no evidence of LTBI. A more detailed overview of the clinical, preparatory and therapeutic details can be viewed in Table VII. Similarly to found regarding TST, there was no significant influence of using DMARDs and steroids on active TB infection.

The TST was not repeated during follow-up in adults with CIA. In patients with JIA, the TST was repeated annually and only one patient became positive after 10 months of the first test, but without epidemiological history. To date, the patient remains asymptomatic and with adequate control of rheumatic disease.

**TABLE VII:** Clinical, propaedeutic and therapeutic details from nine patients with active mycobacterial infections after using tumour necrosis factor (TNF)a blockers

Patient ID	CIA	TST	Prior TNFα blocker	Time of using (months)	Site and treatment of aMI	Diagnosis	Treatment of CIA after aMI	Evolution
1	AS	NR	IFX	3	Lung RIP/6 months	Sputum	NSAID	Good, without recurrence after 18 months
2	AS	NR	IFX	3	Lung RIP/6 months	Sputum	NSAID	Good, without recurrence after 24 months
3	AS	NR	IFX	3	Lung RIP/6 months	Sputum	ETN	Good, without recurrence after 18 months
4	AS	NR	IFX	6	Lung RIPE/6 months	Sputum/HRCT	ETN	Good, without recurrence after 12 months
5	AS	NR	IFX	3	Miliary RIP/9 months	Sputum/HRCT	ETN	Good, without recurrence after 36 months
6	AS	NR	IFX	3	Lung and lymph node RIP/6 months + RIPE/6 months Total time: 12 months	BAL and FNAB	NSAID	Difficult treatment, with lymph node and multi-resistant recurrence
7	RA	NR	IFX	48	Pleural and pancreatic RIP/6 months	Pleura and abdominal CT	RTX	Good, without recurrence after 24 months
8	RA	NR	ADA	25	Lepromatous leprosy	Skin biopsy	GC	Difficult treatment. No recurrence, but with reactional state, needing elevated doses of GC
9	JIA	NR	ADA	24	Lung RIP/6 months	HRCT	ETN	Good, without recurrence after 18 months

ADA: adalimumab; aMI: active mycobacterial infection; AS: ankylosing spondylitis; BAL: bronchoalveolar lavage; CIA: chronic inflammatory arthropathies; CT: computerised tomography; ETN: etanercept; FNAB: fine-needle aspiration biopsy; GC: glucocorticoids; HRCT: high resolution computed tomography of the chest; IFX: infliximab; JIA: juvenile idiopathic arthritis; NR: nonreactive; NSAID: nonsteroidal antiinflammatory drugs; RA: rheumatoid arthritis; RIP: rifampicin, isoniazid, and pyrazinamide; RIPE: rifampicin, isoniazid, pyrazinamide and ethambutol; RTX: rituximab; TST: tuberculin skin test.

## DISCUSSION

Our results showed that the incidence of aMI, especially TB, was elevated in CIA patients with negative evaluation for LTBI at baseline when compared to control group, as well as with the adult Brazilian population and the general paediatric (tabnet.datasus.gov.br/cgi/deftohtm). In addition, the cases were potentially severe and with greater frequency of resistance to treatment, complicating the clinical handling of these patients, including the prescription of GCs and DMARDs, as well as the postponement of the introduction of anti-TNFα agents and its withdrawal at the time of aMI's diagnosis.

According to the British Society for Rheumatology Biologics Registers, the incidence of cases of active TB infection in this scenario was also relevant when compared with the general population and varied between anti-TNFα agents (Dixon et al. 2010). The difference among the agents was also observed in the Spanish registry BIOBADASER. According to this study, monoclonal antibodies were more associated with new cases than against soluble TNFα receptor (Reino et al. 2007). The Brazilian Biological Registry (BiobadaBrasil) reported three cases of TB and one case of tuberculoid leprosy among 1,037 patients included in the first two years (Titton et al. 2011). In our cohort, IFX remained as the main agent associated with aMI, even after adjustments for frequency and exposure time. In addition, it was switched for ETN in active AS patients without recurrence of aMI after 24 months.

The main mechanism to explain the greater rate of TB in this new scenario is that the members of the superfamily of TNFα and the TNFα receptor are important regulators of proliferation, survival, differentiation and apoptosis of immune cells, as well as they play a central role in the initial response of the host to an infectious agent. In mycobacterial infections, they regulate the macrophagic activation, assist the recruitment of cells to the site of infection and promote granuloma formation and maintenance (Flynn et al. 1995, Wallis 2008). Thus, the effect of anti-TNFα therapy may cause disintegration of the granulomatous structure, leading to diffusion of its content and greater chance of active disease (Mack et al. 2009).

In patients with AS, there was a very clear temporal relationship between the initiation of TNFα blockers and the emergence of active infection, stressing that the mechanism of LTBI reactivation was the most involved in these cases. On the other hand, in patients with RA and the child with JIA, the re-exposure to bacillus was the most likely reason, since the symptoms appeared after more than 12 months of therapy. In this sense, epidemiological surveillance, constantly and in each consultation, especially in relation to symptoms and family and professional contacts, is of fundamental importance. In general, the active questioning about these aspects is done just before the introduction of anti-TNFα therapy and not more again during follow-up.

Although the diagnosis of LTBI is difficult, especially in immunocompromised patients, and there are no methods that are considered the gold standard for its adequate characterisation, the TST is the most widely used in clinical practice. However, patients with CIAs, particularly those with RA, present a certain inability to produce an adequate response to the test, even when infected by *M. tuberculosis*, since cellular immune response dependent on the migration of CD4 T-cells, producers of interferon (IFN)g, to the site of inoculation of PPD of*M. tuberculosis*, may be compromised. Currently, this test has been considered inappropriate for the recognition of latent forms in these patients and some countries no longer recommend the TST for LTBI screening (ATS 2000).

In Brazil, Marques et al. (2009a) showed that there is attenuation of response to PPD in patients with RA when compared to HCs (14.6% vs. 33.3%; p = 0.03). A similar finding was found in a study performed in Peru (29% vs. 71%), even with low doses of GCs (Ponce de León et al. 2005). Koker et al. (2007) verified low PPD positivity (29.8%) in patients with RA when compared to AS (65.9%), gout (68.8%) and osteoarthritis (63%). In our sample, TST positivity was significantly more frequent in AS patients than in RA patients at a ratio of 3:1. When compared to HC group, there was no significant difference of the frequency of TST positivity among patients with AS.

It is important to point out that the recommendations suggested by BIOBADASER for LTBI screening were used in the clinical management of patients in this study, with the exception of PPD-Booster (Wallis 2008). However, these tools were not sufficient to identify new cases of aMI in patients with negative LTBI screening at baseline, especially those with AS. Thus, they were somewhat unprotected, since, to date, there is no recommended course of treatment for these cases (nonreactive TST, without epidemiology and normal chest radiography) and, therefore, no form of LTBI prevention is performed.

The development of alternative testing for LTBI identification has been the subject of countless studies. The ideal test should have high sensitivity in all populations at risk, as well as high specificity, regardless of previous bacillus Calmette-Guérin (BCG) vaccination or infection by other mycobacterium.

The recognition of the crucial role of IFNγ on cell response against *M. tuberculosis* has led to the development of trials that evaluate the release of this cytokine [IFNγ release assay (IGRAs)] in the detection of TB infection. IGRAs represent an advance in relation to the TST, since they evaluate the response to peptides that represent proteins specific to *M. tuberculosis*such as the 6 kDa early secretory antigenic target and 10 kDa culture filtrate antigen (Mazurek et al. 2010).

The specificity of IGRAs in immunocompromised patients is 67% against 98.1% in immunocompetent healthy patients. The sensitivity in immunocompromised and healthy patients is 81% and 98%, respectively. The specificity and sensitivity of PPD in this scenario is 49% and 33%, respectively (Diel et al. 2011). However, there are few studies involving patients with CIA (Marques et al. 2009b) or candidates for biologic therapy as psoriasis (Lima et al. 2011) and these are almost nonexistent in children and adolescents with JIA.

The absence of data on prior BCG vaccination, including the scar, represented one of the main limitations of this study. However, to our knowledge, this is the first long-term prospective study conducted in an endemic country with high rate of TB which described the incidence of new cases of aMI in patients, including adults, children and adolescents, with various active CIAs and negative evaluation for LTBI at baseline after starting of TNFα antagonists.

Thus, additional research is needed to define the best strategy to minimise the risk of aMI in patients with CIA after using TNFα blockers, including IGRAs, but also the role and clinical utility of the PPD-Booster phenomenon in predicting the development of active infection by *M. tuberculosis*, especially in countries with high prevalence of the disease, such as Brazil.
